# Bleeding Complications of Anticoagulation Therapy Used in the Treatment of Acute Coronary Syndromes—Review of the Literature

**DOI:** 10.3390/jcm14103391

**Published:** 2025-05-13

**Authors:** Michał Kosowski, Maciej Kocjan, Michalina Mazurkiewicz, Marta Gamrot-Wrzoł, Sabina Ryl, Krzysztof Nowakowski, Jakub Kawecki, Tomasz Kukulski, Damian Kawecki, Beata Morawiec-Migas

**Affiliations:** 1 2nd Department of Cardiology, Faculty of Medical Sciences in Zabrze, Medical University of Silesia, 40-752 Katowice, Poland; maciej.kocjan@onet.eu (M.K.); michalina.liput@gmail.com (M.M.); j.kawecki.jk7@gmail.com (J.K.); tkukulski@sum.edu.pl (T.K.); beata.morawiec@sum.edu.pl (B.M.-M.); 2 Department of Otorhinolaryngology and Oncological Laryngology, Faculty of Medical Sciences in Zabrze, Medical University of Silesia, 40-055 Katowice, Poland; marta.gamrot@gmail.com; 3 Department of Anaesthesiology and Intensive Care, Municipal Hospital in Zabrze-Biskupice, 41-803 Zabrze, Poland; rylsabina@gmail.com; 4 Department of Urology and Urological Oncology in Rybnik, Faculty of Medical Sciences in Zabrze, Academy of Silesia, 40-752 Katowice, Poland

**Keywords:** antiplatelet drugs, heparins, fondaparinux, fibrinolytics, acute coronary syndrome, bleeding complications

## Abstract

Bleeding complications are a significant concern in the management of acute coronary syndromes (ACS). The evidence from clinical trials demonstrates the need for balancing efficacy in reducing ischemic events with safety concerns, as bleeding events adversely affect prognosis and mortality. Pharmacological agents like aspirin, P2Y12 inhibitors (e.g., prasugrel, ticagrelor), glycoprotein IIb/IIIa inhibitors, and heparins are fundamental to ACS treatment but carry varying bleeding risks depending on individual patient profile. Recent advancements in risk stratification tools have enabled tailored approaches to dual antiplatelet therapy (DAPT), optimizing its duration based on bleeding and thrombotic risks. Further Emerging therapies, including shortened DAPT protocols and P2Y12 inhibitor monotherapy, have shown promise in minimizing bleeding while maintaining clinical efficacy. The findings underscore the importance of personalized antithrombotic regimens in ACS management, emphasizing precise risk assessment to enhance outcomes and mitigate adverse events. This review examines the mechanisms, risk factors, and strategies to mitigate bleeding associated with anticoagulant and antiplatelet therapies in ACS.

## 1. Introduction

Acute coronary syndrome (ACS), often the first appearance of coronary artery disease (CAD), is a life-threatening condition and requires accurate and advanced treatment in order to restore optimal blood flow in the infarct-related coronary artery. Alongside invasive pharmacological efforts put on treating the ACS and restoring the blood flow in coronary arteries expose the patient to a higher risk of bleeding in the acute phase, but also in the long term. There is some data showing that bleeding events worsen the outcome, so the current strategy for the management of ACS is to minimize adverse ischemic events and decrease the risk of bleeding. Therefore, appropriate anticoagulant therapy should be individualized in terms of quality and quantity based on comorbidities, clinical status, and optimal assessment of the risk and early signs of bleeding associated with this specific therapy, including life-threatening bleeding [1,2].

All drugs affecting the coagulation system recommended for the treatment of ACS are summarized in Table 1.

This review focuses on understanding the mechanisms of action and clinical characteristics of anticoagulant and antithrombotic therapy used in the treatment of ACS, the methods of assessing the bleeding risk and potential ways to reduce it.

## 2. Antiplatelet Drugs

### 2.1. Mechanism of Action

**Figure 1:**
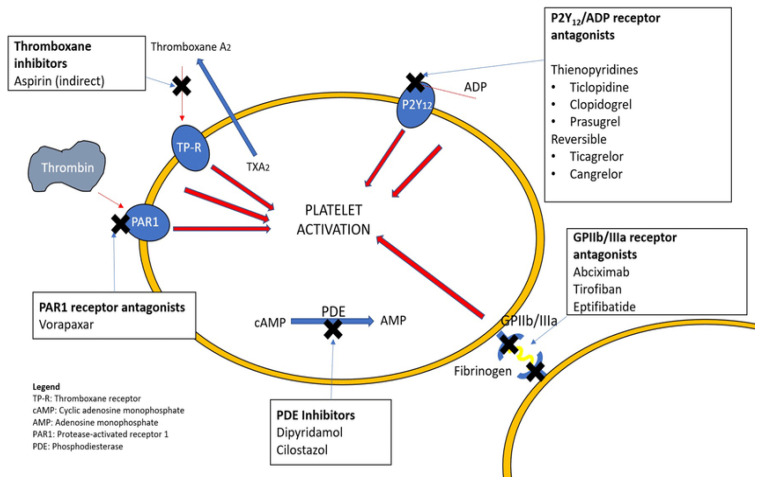
Common antiplatelets and their mechanism of action [3].

Antiplatelet drugs are used with class I of recommendation in the daily practice for the treatment of ACS. The first antiplatelet drug introduced into practice was acetylsalicylic acid, which is a cyclooxygenase inhibitor. Antiplatelet agents might be divided according to the way of administration into oral and parenteral agents or by the mechanism of action (Figure 1) into the following:

- Platelet aggregation inhibitors:
  ○ Aspirin and related cyclooxygenase inhibitors
  ○ Oral thienopyridines, which are P2Y12 inhibitors such as clopidogrel, ticagrelor, ticlopidine, and prasugrel
- Glycoprotein platelet inhibitors (e.g., abciximab, eptifibatide, tirofiban)
- Protease-activated receptor-1 antagonists (e.g., vorapaxar)
- Miscellaneous (e.g., dipyridamole—a nucleoside transport inhibitor and phosphodiesterase type 3 [PDE3] inhibitor, cilostazol—a PDE3 inhibitor)

### 2.2. Risk of Bleeding

**Figure 2:**
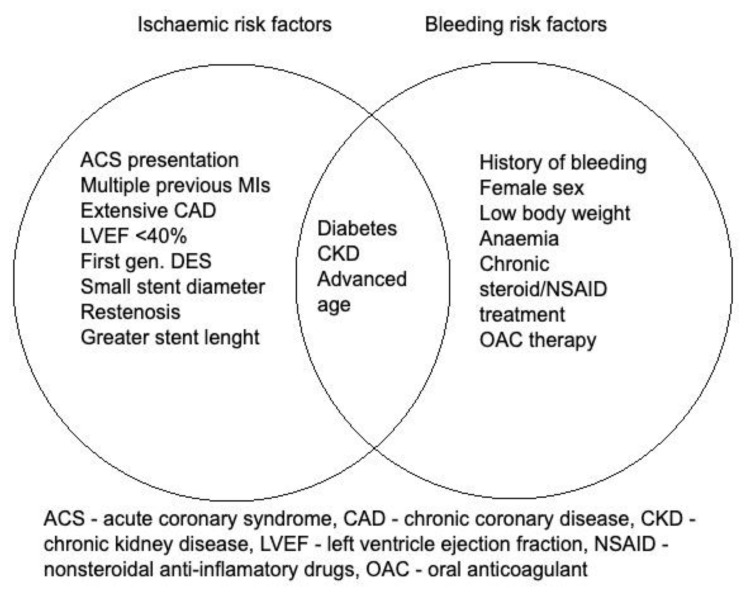
Risk factors of bleeding and ischemic events.

Before starting taking antiplatelet agents, the patient should undergo an assessment for bleeding risk. Evaluation of risk factors for ischemia and bleeding is an integral part of determining the optimal duration of dual antiplatelet therapy (DAPT), which is recommended in every case of patients after percutaneous coronary intervention (PCI). Factors that could increase the first of them include advanced age, exacerbation of ACS, diabetes, and LVEF < 40%, while conversely, the risk of bleeding increases previous bleeding, current anticoagulation, female sex, or steroid/NSAID use (Figure 2).

Over the past 10 years, multiple risk scores have been developed and validated to properly identify bleeding risk in patients after PCI. When planning treatment, we have to consider the patient’s risk factors for bleeding, the choice of treatment method and the selection of an appropriate antiplatelet drug. In everyday clinical practice, we can use scales that were developed based on multicenter clinical trials.

It is worth noting that each bleeding risk score takes into account a few specific risk factors, and none of those consider just a single factor. Due to these scales, we are able to identify patients with an increased likelihood of bleeding complications in the long-term observation [4].

The PRECISE-DAPT score is a five-item risk score that gives a unified tool for predicting bleeding during DAPT. The study shows that in a total of 21,963 person-years of follow-up, out-of-hospital TIMI major or minor bleeding occurred in 218 patients (1-year incidence 12.5 per 1000 patients [5].

CREDO-Kyoto results showed moderate accuracy in stratifying the risk of thrombosis and bleeding. However, to a large extent, patients at high thrombotic risk also had a high bleeding risk [6].

BleeMACS registry will help to establish a risk score to predict the occurrence of major bleeding in patients receiving DAPT after ACS [7].

The PARIS Thrombotic Risk Score may help to identify patients at higher risk of mortality and major adverse cardiac and cerebrovascular events (MACCE). Regarding thrombotic risk, it has modest, long-term nonhospital prognostic value for mortality and MACCE in patients undergoing PCI. The prognostic value of mortality is greater than MACCE [8].

#### 2.2.1. Oral Antiplatelet Drugs

Acetylsalicylic acid (ASA) is one of the most frequently used drugs in the world [9]. However, the use of aspirin in low doses (75–325 mg/d) is associated with several side effects; the most clinically significant are serious extracranial and gastrointestinal bleeding. The risk of bleeding into the upper gastrointestinal tract when using aspirin in monotherapy is relatively low and amounts to 0.05–0.36% [10]. When it comes to bleeding in the lower gastrointestinal tract, the risk is even lower and amounts to 0.048–0.074% [10].

In the group of patients with ACS and after PCI, it is of utmost importance to radically inhibit platelet aggregation, which is recommended by DAPT with ASA and a P2Y12 receptor inhibitor. Such a strategy obviously improves short and long-term prognosis in terms of ischemic events but also increases the risk of hemorrhagic complications, which in turn worsens the prognosis and increases mortality in these patients.

To minimize the risk of bleeding currently while maintaining a low thrombotic risk, various models of DAPT duration strategies and the use of a PY12 inhibitor as monotherapy are also being considered. Over the past decade, discussions about the optimal duration of DAPT have shifted dramatically from a recommendation of 6/12 months to a more flexible and dynamic protocol that takes into account various factors, including CAD status and bleeding risk. Typically, in patients without an increased risk of bleeding, DAPT is recommended for 1 year in ACS and 6 months in stable CAD, followed by lifelong aspirin monotherapy. In those who have completed standard DAPT without severe bleeding, continuation of DAPT for longer than 1 year may be considered in patients at high risk of recurrent adverse cardiovascular events (post-infarction patients). The evidence for a shorter DAPT duration of 6 months in patients undergoing PCI for stable CAD was based on two landmark studies in the early 2010s.

The EXCELLENT study enrolled 1443 patients. Two groups of patients were distinguished. Treated DAPT (ASA + clopidogrel) for 6 vs. 12 months in stable CAD. The primary endpoint was cardiac death, myocardial infarction, or ischemia-induced target vessel revascularization. After 12 months of observation, it was noted in 4.8% in the 6-month group and 4.3% in the 12-month DAPT group [11].

In the PRODIGY 2013 study, patients were randomly assigned to two groups, receiving DAPT for 6 and 24 months (ASA + clopidogrel), and the primary endpoint included death, myocardial infarction and central nervous system ischemic event, including stroke. There was no statistically significant difference in the occurrence of the primary composite endpoint after 24 months of follow-up (10.1% vs. 10.0%), but importantly, shorter DAPT was associated with a lower risk of major bleeding (1.9% vs. 3.4%) [12]. It is worth noting, however, that, as the authors write, this exploratory study is underpowered and should be considered hypothesis-generating only.

A separate, very important problem is the use of antiplatelet drugs in combination with anticoagulants in patients who require constant anticoagulation, such as in atrial fibrillation. The first prospective study comparing the efficacy and safety of dual therapy (P2Y12 inhibitor + vitamin K antagonist) and triple therapy (aspirin + P2Y12 inhibitor + vitamin K antagonist) was the WOEST trial. It showed that not only is the frequency of bleeding complications lower in the group of patients receiving dual therapy, but also the frequency of cardiovascular complications is lower [13]. However, these results should be approached with caution because the ACS group was underrepresented in the study, and WOEST was not powered to assess these outcomes.

Currently, the dominant anticoagulants in the treatment of patients are not vitamin K antagonists but rather direct oral anticoagulants (DOACs). Rivaroxaban was the first to be tested in a group of patients with atrial fibrillation who required PCI. The PIONEER-AF PCI study demonstrated the superiority of reduced-dose rivaroxaban over warfarin as an adjunct to antiplatelet therapy in reducing the risk of bleeding [14]. More importantly, there was no difference between groups in the risk of major adverse cardiovascular events. Identical observations for the remaining DOACs were obtained in the RE-DUAL trial for dabigatran and in the AUGUSTUS trial for apixaban [15,16]. The conclusions from all the above-mentioned studies in order to reduce the risk of bleeding promote the approach of maximally shortening the time of using triple therapy in favor of double therapy (P2Y12 inhibitor + oral anticoagulant) and additionally show the higher safety of DOACs over vitamin K antagonists.

#### 2.2.2. Intravenous Antiplatelet Drugs—Review of Clinical Trials

Antiplatelet drugs are used parenterally much less frequently than orally. This is due to the fact that their use is in a selected group of patients, which is the treatment of ACSs. Here, we distinguish the P2Y12 inhibitor—cangrelor—and glycoprotein IIb/IIIa inhibitors—abciximab, eptifibatide and tirofiban. Their major adverse effect is bleeding. The balance between safety and effectiveness is crucial for proper use.

Cangrelor is the only P2Y12 receptor antagonist used intravenously. Cangrelor is characterized by a very rapid onset of action and directly proportional, dose-dependent pharmacokinetics. Inhibits platelets by over 90%. Its half-life is very short, and it is a few minutes, and the antiplatelet effect disappears quickly—within 1–1.5 h [17].

We have available results from three large clinical trials that were placebo-controlled and randomized. They demonstrate the effectiveness and safety of cangrelor in a wide range of patients with coronary artery disease treated with coronary angioplasty: CHAMPION PLATFORM, CHAMPION PCI and CHAMPION PHOENIX [18,19,20].

The CHAMPION PLATFORM trial recruited 5362 patients requiring PCI mainly for non-ST-segment elevation myocardial infarction (NSTEMI) (59.4%) but also unstable angina (35.4%). Patients with stable angina (5.2%) were initially qualified before the inclusion criteria were changed. The primary endpoint—coincidence of death and myocardial infarction, or ischemic revascularization within 48 h after PCI, was lower in the cangrelor group than in the placebo group, but this difference was not significant. The incidence of stent thrombosis was significantly lower in the cangrelor group at 48 h and 30 days. All-cause mortality was significantly lower in patients treated with cangrelor at 48 h but not at 30 days. The bleeding complications did not differ significantly between these two groups according to TIMI and GUSTO criteria. However, based on ACUITY criteria, the bleeding rate was significantly higher in the cangrelor group. The difference is only due to the presence of hematomas and not to the occurrence of major bleeding.

The subsequent CHAMPION PCI study enrolled a total of 8877 patients treated with percutaneous coronary angioplasty for stable angina (15.0%), unstable angina (24.6%), NSTEMI (49.2%), or STEMI (11.2%; *n* = 996). The primary endpoint of death from any cause, myocardial infarction, or ischemic revascularization at 48 h occurred in similar proportions in both study groups: the experimental one (cangrelor with clopidogrel) and the active control group (placebo and clopidogrel). There were no significant differences between those two for any single efficacy endpoint at 48 h. According to ACUITY and GUSTO criteria, minor but not major bleeding occurred more frequently in the cangrelor group. According to TIMI criteria, there was no increased severity of bleeding, regardless of the type of bleeding [19]. However, both the CHAMPION PLATFORM and CHAMPION PCI studies had a problem with the definition of ACS used in them, which ultimately led to the conclusions drawn from them not having sufficient power. Only in the CHAMPION PHOENIX study was a definition based on the second universal definition of MI used. The CHAMPION PHOENIX trial was designed to assess whether cangrelor reduces ischemic complications associated with coronary angioplasty. A total of 10,942 patients requiring PCI for stable angina (56.1%), non-ST-segment elevation ACS (NSTE-ACS) (25.7%), or STEMI (18.2%) were enrolled and received intravenous cangrelor or placebo. The rate of the primary composite endpoint of all-cause death, myocardial infarction, ischemia-induced revascularization, or stent thrombosis within 48 h was significantly lower with cangrelor than with clopidogrel. In addition to the reduction in stent thrombosis, the benefit of cangrelor in CHAMPION PHOENIX was primarily attributed to the reduction in the incidence of AMI. The 22% reduction in the odds of an ischemic event in patients treated with cangrelor was not associated with a significant increase in the risk of major bleeding or transfusion compared with patients treated with clopidogrel. There was an increase in bleeding in patients treated with cangrelor, which would be expected for a more potent antiplatelet drug [20].

Glycoprotein IIb/IIIa receptor antagonists are a class of drugs used to treat patients with ACS undergoing PCI. Three glycoprotein receptor inhibitors (GPIs), abciximab, eptifibatide, and tirofiban, are now commonly used. Eptifibatide and tirofiban are small-molecule GP IIb/IIIa inhibitors, whereas abciximab is a humanized fragment of a murine monoclonal antibody [21].

The most potent and best-known antagonist of the GP IIb/IIIa receptor is abciximab. Abciximab binds with high affinity to the GP IIb/IIIa receptor, resulting in slow dissociation kinetics and a long platelet half-life despite the short plasma half-life of the drug. Thus, platelet inhibition by abciximab lasts approximately 48 h after drug discontinuation [22]. There have been several clinical trials comparing the effectiveness of abciximab versus placebo.

In the first study (EPIC) of 2099 high-risk ischemic PCI patients receiving abciximab by intravenous infusion, a significant 35% reduction in the primary endpoint at 30 days was observed compared with placebo (12.8% vs. 8.3%, *p* = 0.008). There was a significant increase in both major bleeding and transfusion events in patients receiving bolus and infusion of abciximab, particularly site bleeding from coronary artery bypass graft and vascular access [23].

The EPILOG study evaluated a lower-risk population and compared 3 groups according to the dose of low-molecular-weight heparin used. A total of 2792 patients were enrolled in the study, and the study was stopped after an interim analysis showed a >50% reduction in the risk of the primary efficacy endpoint in the 2 abciximab treatment groups. The incidence of the primary endpoint was 11.7% in the placebo group vs. 5.2% in the abciximab plus low-dose heparin group and 5.4% in the abciximab plus standard-dose heparin group. The rate of major bleeding was lower in the abciximab plus low-dose heparin group (2.0%) compared with patients in the abciximab plus standard-dose heparin group (3.5%) and the placebo plus standard-dose heparin group (3.1%), although the differences were not statistically significant [24].

The EPISTENT trial included 2399 patients undergoing PCI. Patients were randomly assigned to receive a stent plus abciximab, a stent plus placebo, or balloon angioplasty plus abciximab. Patients receiving a stent had a 52% reduction in the risk of the primary endpoint in the abciximab group compared with placebo (5.3% vs. 10.8%). Patients in the balloon angioplasty plus abciximab group also had a lower rate of the primary endpoint than the stent plus placebo group (6.9% vs. 10.8%). Major bleeding occurred in 2.2% of patients in the stent plus placebo group, 1.5% in the stent plus abciximab group, and 1.4% in the balloon angioplasty plus abciximab group. These differences were not statistically significant [25].

The CAPTURE study evaluated the efficacy and safety of abciximab in patients with unstable angina, defined as recurrent ischemia, despite treatment with heparin and nitrates, and the benefit of abciximab given as pretreatment before the procedure. A total of 1265 patients were included and scheduled to receive abciximab or placebo for 18 to 24 h before PCI and 1 h after the procedure. The primary endpoint was 11.3% in the abciximab group and 15.9% in the placebo group. There was an increase in the rate of major bleeding in the abciximab group (3.8% vs. 1.9%) [26].

For eptifibatide, the largest study to date is the PURSUIT study. A total of 10,948 patients were enrolled in the registry. The study showed that the use of eptifibatide significantly reduced the mortality and incidence of myocardial infarction in the group of patients undergoing coronary artery bypass grafting within 72 h of randomization (16.2% vs. 30.8% in the placebo group). The beneficial effect of the drug was maintained on days 7 and 30 of follow-up [27].

As for the third drug, tirofiban, we have the PRISM study available here, which included 3232 patients with unstable angina who were treated with tirofiban or heparin. During the 48-h observation, tirofiban was shown to be superior in reducing the frequency of a clinical event defined as death, myocardial infarction or recurrent ischemia (3.8% vs. 5.6% in the heparin group). However, the differences between the groups assessed 30 days after the start of treatment turned out to be statistically insignificant.

In the modified version of the PRISM study (PRISM-PLUS), the effect of tirofiban and/or heparin administration in patients with unstable angina pectoris after PCI was analyzed. It was shown that the concomitant administration of tirofiban and heparin was associated with a significantly lower incidence of composite clinical events during the 7-day observation period compared with heparin monotherapy (12.9% vs. 17.9%). This effect was also observed in the analysis conducted one month after randomization (5.9% vs. 10.2% in the heparin group) [28]. However, when analyzing this study, attention should be paid to two serious limitations. The first is that it was a post-hoc analysis, and the size of the group is not very large. The second limitation is related to the period of enrollment of patients in the PRISM PLUS study, which took place in the years 1994–1996, i.e., before the widespread use of troponin concentration testing, which meant that the entire process of diagnosing myocardial infarction in those years was different, and this significantly influenced the process of enrollment.

The ESPRIT trial included a large group of 2064 patients who randomly received eptifibatide or placebo during PCI. All were routinely taking aspirin, thienopyridine, and heparin. The primary composite endpoint of the trial included death, myocardial infarction, target vessel revascularization, and the need for rescue administration of eptifibatide due to clinical or angiographic complications, assessed combined within 48 h of randomization. The trial was stopped early because the clinical efficacy endpoints for eptifibatide therapy had been met. The primary endpoint (assessed at 48 h) occurred in 6.6% of eptifibatide-treated patients and in 10.5% of placebo-treated patients. These favorable outcomes were maintained at 30 days, 6 months, and 1 year after randomization. The incidence of the composite endpoint at 12 months was 17.5% in the eptifibatide group and 22.1% in the placebo group. Despite the high dose of eptifibatide, no effect of active treatment on the incidence of bleeding complications was observed. Severe bleeding, defined by GUSTO criteria, occurred in 0.7% of eptifibatide-treated patients and 0.5% of placebo-treated patients [29].

The IMPACT II trial included 4010 patients undergoing elective, urgent, or emergency coronary intervention. Patients were assigned to one of three treatment regimens: placebo or a variable dose regimen of eptifibatide (135/0.5 vs. 135/0.75). The primary endpoint was the 30-day composite of death, myocardial infarction, unplanned surgical or repeat percutaneous revascularization, or coronary stent implantation for emergency closure. At day 30, the composite endpoint occurred in 151 (11.4%) patients in the placebo group vs. 124 (9.2%) in the eptifibatide 135/0.5 group and 132 (9.9%) in the eptifibatide 135/0.75 group. Analysis of the treatment received showed that the 135/0.5 regimen resulted in a significant reduction in the composite endpoint (11.6 vs. 9.1%), but the 135/0.75 regimen resulted in a less significant reduction (11.6 vs. 10.0%). Eptifibatide treatment did not increase the incidence of major bleeding or transfusion [30].

The largest clinical trial comparing GP IIb/IIIa inhibitors is the TARGET registry, which compared abciximab with tirofiban to demonstrate the noninferiority of tirofiban. A total of 5308 patients undergoing PCI were assigned to receive abciximab or tirofiban. All patients received heparin and aspirin, with a loading dose of clopidogrel when available. The primary endpoint occurred at a higher rate in the tirofiban group than in the abciximab group (7.6% vs. 6.0%), indicating that the two drugs were not equivalent and that abciximab was superior to tirofiban. The rate of major bleeding or transfusion was similar in the two groups (0.9% vs. 0.7%) [31]. 

We have summarized all studies on intravenous antiplatelet drugs in Table 2.

### 2.3. Special Group of Patients with High Bleeding Risk

According to current ESC guidelines, patients at increased bleeding risk with stable CAD should be continued with DAPT for 3 months, followed by aspirin monotherapy. However, in people at very high risk of bleeding, shortening DAPT to 1 month may be considered. In the group of patients after ACS, in patients at high risk of bleeding, DAPT should be continued for 6 months, and then aspirin monotherapy should be used. However, in patients at very high risk of bleeding who experience any of these symptoms, shortening DAPT to 1–3 months, followed by monotherapy with a P2Y12 receptor inhibitor, may be considered.

The first studies confirming the shortening of the DAPT duration—3 months—were RESET OPTIMIZE. The RESET trial randomized 2117 patients to two groups, namely 3 and 12 months (DAPT aspirin + clopidogrel), and found no difference in the primary composite endpoint of all-cause mortality, myocardial infarction or ST (0.8 vs. 0.3%; *p* = 0.48). Of these patients, 44.28% developed ACS. Analysis of ACS patients in this group revealed an increased rate of the primary composite endpoint in patients in the shortened DAPT group but without statistical significance (*p* = 0.158). OPTIMIZE, on the other hand, randomly recruited 3119 patients to the same groups of 3 and 12 months of DAPT (ASA + clopidogrel). The results of this study were similar, but the primary endpoint of net adverse clinical events (NACE) occurred in 6.0% of the first group and 5.8% in the second group [32,33].

In recent years, further evidence has emerged confirming the effectiveness and safety of shortening DAPT therapy to 3 months, followed by monotherapy with a P2Y12 receptor inhibitor.

The SMART-CHOICE trial randomized 2994 patients to 3 and 12 months of DAPT with aspirin and a P2Y12 receptor inhibitor followed by only a P2Y12 receptor inhibitor, and the results were similar, with no significant difference in NACE (4.5 vs. 5.6% and a significant reduction in the incidence of major bleeding (2.0 vs. 4%) [34].

TICO and Mehran et al.’s study compared 3- and 12-month DAPT with aspirin and ticagrelor, followed by ticagrelor monotherapy. Mehran et al. trial showed no significant difference in net adverse clinical events (NACE) (3.9 vs. 3.9%), while the TICO trial showed that short DAPT reduced the incidence of NACE (3.9 vs. 5.9%). Both cases showed a significant reduction in the incidence of major bleeding in the short DAPT group (Mehran et al., 4.0 vs. 7.1% and TICO, 1.7 vs. 3.0%) [35,36].

The most recently published study provides evidence for an even more shortened 1-month DAPT treatment after PCI in certain patient populations [37]. This is particularly important in patients at very high risk of bleeding. (in patients with AF requiring anticoagulant treatment) or in those who require emergency surgical intervention due to other diseases.

The MASTER-DAPT study, which compares 1-month and 3-month DAPT and, significantly, the first group is not inferior in terms of the occurrence of NACE (7.5% vs. 7.7%), and at the same time, proves a statistically significant reduction in the risk of bleeding (6.4% vs. 9.4%) [38].

There are few other studies directly comparing these two short-term DAPT protocols, making this an area where future research can be used to further optimize DAPT management after PCI.

Additionally, there are not many studies comparing these two short-term DAPT protocols, making this an area where future research could be used to further optimize DAPT treatment after PCI.

Another study focused on which drug to leave as monotherapy after DAPT. Both the one-month DAPT trial and the XIENCE short DAPT program showed similar benefits with short-term DAPT followed by aspirin monotherapy. Major bleeding occurred statistically less frequently (One-month DAPT 1.7% vs. 2.5% and in the Xience Short DAPT groups 2.2% vs. 6.3% and 2.2% vs. 4.5%, respectively). When it comes to comparing mortality in both groups, statistically, the values were close to each other [39,40].

Studies such as STOPDAPT-2, GLOBAL LEADERS (1 vs. 12 months) and Sidney-2-Collaboration (1 vs. 3 months) compared the effects of shortening DAPT to 1 month and maintaining P2Y12 inhibitor monotherapy. All these trials showed statistically fewer bleeding complications while maintaining a comparable risk of NACE, major adverse cardiovascular events (MACE) and mortality [41].

In 2023, a very important meta-analysis of 11 clinical trials was published, which clearly showed the superiority of DAPT shortened to 1 or 3 months in the group of patients at high risk of bleeding. These studies consistently show that in this group of patients, shortened DAPT resulted in a lower incidence of major bleeding but also lower overall cardiovascular mortality. Furthermore, it is important to note that shortened DAPT did not result in a higher incidence of recurrent cardiovascular events or stent thrombosis. However, there is currently no consensus and no sufficient evidence to support the superiority of DAPT shortened to 1 month over 3 months of treatment [42].

It is also worth mentioning that a special group of patients with an initially higher risk of bleeding are women. Less than a year ago, a meta-analysis was published summarizing the influence of gender on the occurrence of both hemorrhagic and cardiovascular events in patients taking antiplatelet drugs. This analysis, which included 22 clinical trials, showed that female gender was associated with a significantly higher risk of both bleeding and major cardiovascular and cerebrovascular events. Sub-group analysis additionally showed that a higher risk of bleeding in women is associated with the use of newer antiplatelet drugs such as prasugrel or ticagrelor. Moreover, shortening DAPT or selecting short-term DAPT followed by P2Y12 inhibitors monotherapy in the treatment regimen did not reduce the risk of bleeding in women, while such a relationship could be observed in the group of men [43].

Over the past decade, opinion on the optimal duration of DAPT has changed significantly from an inflexible recommendation of 6–12 months to a more individually tailored approach that takes into account various risk factors, including the severity of CAD and bleeding risk. Scientific evidence strongly favors short-term DAPT of 1–3 months in selected patients, and ESC guidelines are being progressively updated. However, further studies are necessary to compare the effectiveness and, above all, the safety of 1- and 3-month DAPT and aspirin after DAPT compared to P2Y12 receptor inhibitor monotherapy. On the other hand, future work may also provide new data regarding the indications for long-term DAPT beyond the standard 12 months in patients at low risk of bleeding [44,45].

## 3. Fibrinolytic Drugs

### 3.1. Mechanism of Action

Fibrinolytic drugs are used in situations where embolism occurs in blood vessels [46]. Due to their mechanism of action, they can be divided into plasminogen activators, drugs acting indirectly, and direct fibrinolytic drugs [47]. Plasminogen activators are a group of serine proteases whose mechanism of action is the direct activation of plasminogen to plasmin, which has fibrinolytic properties. They are divided into three generations, which differ not in their specificity of action but primarily in their pharmacokinetics, which directly influence the risk of bleeding complications [48]. The action of direct fibrinolytics skip the plasminogen activation phase. These drugs are proteolytic enzymes that directly degrade fibrin. This mechanism of action means that they not only have a stronger effect than plasminogen activators but also cause fewer bleeding complications [49]. Table 3 presents representatives of individual groups of fibrinolytic drugs along with their mechanism of action.

### 3.2. Epidemiology of Bleeding Adverse Events After Using Fibrinolytics

The history of the use of fibrinolytic drugs from the group of plasminogen activators began in the 1930s with the discovery of streptokinase [61]. Over the course of several years, this drug has been repeatedly described by various authors as potentially effective in removing blockages forming inside blood vessels [62,63]. However, it was only 20 years later, at the turn of the 1950s and 1960s, that studies appeared proving that the supply of this drug significantly reduces the mortality rate of patients with ACS [64], and in 1959, Ruegsegger and his team proved that it is related to the dissolution of embolisms in coronary arteries [65]. In addition to the positive impact on the survival of patients treated with streptokinase, side effects related to its administration were also observed from the very beginning. The first large clinical trial on this issue was the GISSI-2 study, which compared not only the effectiveness of alteplase and streptokinase in the treatment of ACS but also assessed the safety of these drugs. The authors of this study noted the relatively high safety of their use, as episodes of serious bleeding in patients treated with alteplase occurred only in 0.5% of cases and with streptokinase in 1%. Of course, more and less severe bleeding was observed, but in the case of both drugs, the incidence did not exceed 10% [66].

Although plasminogen activators were originally used primarily in the treatment of patients diagnosed with myocardial infarction, nowadays, due to the wide availability of hemodynamic laboratories, their role in the treatment of this disease is marginal. For this reason, the number of studies assessing their safety in this group of patients is relatively small. Available research, however, indicates that the use of fibrinolytic drugs is undoubtedly associated with a higher risk of hemorrhagic complications compared to percutaneous angioplasty and especially a higher risk of hemorrhagic stroke [67]. Factors that directly influence this risk include the patient’s age, the time from the onset of symptoms to the administration of a fibrinolytic drug, and the location and extent of necrosis [68].

All these data lead to the conclusion that the use of therapy with fibrinolytic drugs from the group of plasminogen activators is relatively safe, provided that the indications and contraindications to its administration are properly assessed.

The second group of fibrinolytic drugs discussed in the introduction, i.e., directly acting fibrinolytics, is assumed to be associated with a lower risk of bleeding complications, which is directly related to their mechanism of action. At the moment, due to the fact that they are not used in the treatment of patients, there are no studies that would clearly prove their greater safety compared to the group of plasminogen activators.

### 3.3. Methods of Reducing the Risk of Bleeding in Patients Undergoing Fibrinolysis

Due to the mechanism of action of fibrinolytic drugs, their pharmacokinetics and the lack of drugs that reverse their action, reducing the risk of bleeding is based primarily on the correct qualification of patients for thrombolysis. In the context of ACS, it is worth emphasizing that fibrinolysis is indicated only in the group of patients who suffer from ST-segment elevation infarction, and it is not possible to perform PCI within 120 min of its diagnosis [69]. Equally important in terms of risk reduction is the assessment of whether the patient has any absolute contraindications to the supply of fibrinolytic drugs, which are based on both clinical information and laboratory test results and are common for all cases where fibrinolysis is indicated [70]. The full list of contraindications can be found in Table 4.

Alternative or specific maneuvers as a way to reduce the risk of hemorrhagic complications were investigated In the past, among them the possibility of using low-dose fibrinolytic therapy. Due to its low therapeutic effectiveness, it has never entered clinical practice [71]. Similarly, the possibility of targeted drug delivery to the vessel containing embolic material using intravascular catheters was investigated.

For this reason, currently, only the correct qualifications of patients for thrombolytic treatment based on the above-mentioned indications and contraindications allow for a reduction in the incidence of the hemorrhagic complications described above.

## 4. Heparins

### 4.1. Mechanism of Action

Drugs belonging to the heparin group can be divided into two groups: unfractionated heparin (UFH) and low molecular weight heparins (LMWH). The mechanism of action of UFH and LMWH is not completely the same, which is largely due to differences in their molecular structure.

They are composed of repeating disaccharide units (iduronic acid/glucuronic acid-glucosamine), but UFH is characterized by a larger number of these units in its structure and, therefore, a higher molecular weight, which means that they preferentially bind antithrombin with thrombin (clotting factor IIa). Due to their lower molecular weight, LMWHs have a lower ability to inhibit factor IIa, but they have very strong activity against factor Xa, inhibiting the coagulation system at a higher level [72,73].

The basic indications for the use of UFH are deep vein thrombosis (DVT), pulmonary embolism (PE) and thromboprophylaxis in AF, but this drug is also widely used in off-label indications like in the case of patients with ACS during PCI [74].

Among the numerous indications for the use of LMWH, the most important is the prevention of DVT, treatment of venous thrombosis, PE, myocardial infarction with ST-segment elevation, unstable angina or prevention of clotting in extracorporeal circuits [75].

When considering the mechanism of action of both UFH and LMWH, it should be remembered that, apart from the basic mechanism of binding to coagulation factors through antithrombin, heparins, due to the accumulation of a negative charge on their molecules, interact very strongly with positively charged molecules found in biological membranes and plasma. This phenomenon not only makes their pharmacokinetics unstable but also causes complications such as heparin-induced thrombocytopenia (HIT). These effects are much stronger in the case of unfractionated heparin [76].

**Figure 3:**
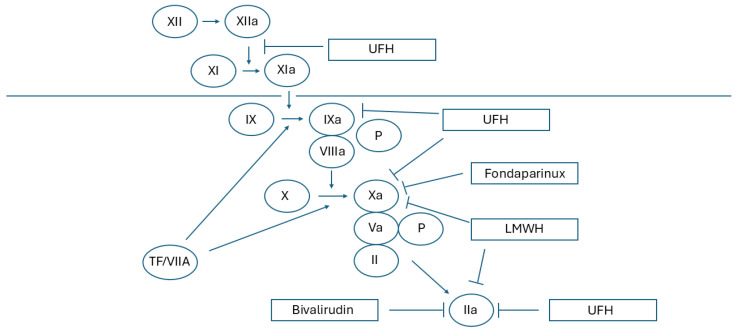
Mechanism of action for heparins.

The molecule resulting from the evolution of heparin, fondaparinux, is devoid of the phenomena discussed above. It consists only of a pentasaccharide sequence, which allows it to selectively bind to the antithrombin molecule and increase its factor Xa-inhibiting activity without affecting thrombin [77]. Due to this mechanism of action, it has 7 times stronger anticoagulant effects compared to LMWH while also being characterized by more stable pharmacokinetics and a lower incidence of complications [78]. It is also worth noting that, due to the same mechanism of action as in the case of LMWH, the scope of indications for its use coincides with the scope typical for heparins. The mechanism of action of heparins is presented in Figure 3.

### 4.2. Risk of Bleeding

Due to the fact that in the case of ACSs, heparins and their derivatives are used on a short-term basis, most often only periprocedural, there are not many scientific reports in the literature assessing the safety of their use because bleeding complications are relatively rare with such a short period of their administration. Additionally, it should be noted that due to the need for parallel administration of antiplatelet drugs in this group of patients, it is difficult to conclude whether they are the direct cause of this complication.

There have been several comparative studies comparing the safety of using UFH, LMWH and bivalirudin, but all of them indicated that there are no statistically significant differences between these drugs in the context of the occurrence of bleeding complications in the group of patients to whom they were administered due to the diagnosis of ACS [79,80,81,82].

Comparing these drugs with fondaparinux is completely different. Due to the very specific mechanism of action described above, it can be expected that its use will be associated with a lower risk of complications. Three meta-analyses from 2016, 2017 and 2019 unanimously indicated that compared to LMWH, the use of fondaparinux is associated with a lower risk of bleeding complications [78,83,84].

## 5. Prevention of Bleeding Complications After Anticoagulation Therapy

In daily clinical practice, the decision regarding the type of anticoagulant therapy in individual cases is still based more on the physician’s subjective opinion than on objective evidence. This is due to the lack of clear data from clinical trials. The best way to prevent bleeding is to properly assess the bleeding risk before starting anticoagulation therapy. Since the release of the 2020 ESC guidelines, the most frequently used tool to assess this risk of bleeding is the HAS-BLED score, especially in the group of patients who require constant anticoagulant therapy due to atrial fibrillation [85]. In the case of patients after ACS, it is worth remembering another scale used to estimate the risk of bleeding in this group of patients, which is the CRUSADE score [86]. However, we have other scales that can help in the assessment of this group of patients [87]. Table 5 also presents the most frequently used scales for assessing the risk of bleeding and the risk factors that are taken into account.

Patients who are at higher risk of bleeding still pose the greatest challenge when making decisions about anticoagulant treatment. Especially since the risk of thromboembolic events in this group is often higher than in the general population [88]. In this situation, the best way to reduce the risk of bleeding seems to be to limit those modifiable risk factors (e.g., hypertension), which, according to the scales presented above, increase it [89]. Furthermore, it should be noted that the bleeding risk may change over time, and the assessment should consider its dynamic changes.

In relation to ACS patients treated with PCI, one of the risk factors for periprocedural bleeding that is worth considering is the selection of appropriate vascular access. The LEADERS FREE trial, which included 2432 patients at high risk of bleeding, showed that there was a statistically significant reduction in major bleeding incidents in the group of patients who had chosen the trans-radial access compared to the transfemoral access. However, most of these events in this population are unrelated to vascular access [90]. The same data come from the RIVA-PCI trial, which included patients at high risk of bleeding with concomitant atrial fibrillation [91]. The conclusions from these studies leave no doubt that the preferred vascular access, especially in patients at high risk of bleeding, should be trans-radial access.

Nowadays, when more and more specific reversal agents such as protamine, idarucizumab or andexenet-alfa are being used, it may seem that the use of anticoagulant drugs for which we have a specific antidote is associated with greater safety for patients. However, it should be remembered that there is no data from RCTs that would indicate the superiority of using specific antidotes over supportive care, which is used in the case of bleeding after each anticoagulant [92].

In relation to antiplatelet therapy, in the case of which we cannot afford to supply a reversal agent, the only method to reduce the risk of bleeding, apart from its proper estimation and selection of appropriate therapy, is also the use of prophylactic drugs. Such drugs include proton pump inhibitors (PPI). One of the most important clinical trials evaluating the effectiveness of combining DAPT with PPI is the COGENT study. It showed high effectiveness in reducing the risk of gastrointestinal bleeding incidents without increasing the cardiovascular risk in the group of patients taking omeprazole with DAPT [93]. More importantly, post-hoc analysis in high-risk cardiovascular patients supports the use of clopidogrel and PPIs to reduce bleeding risk without increasing the risk for cardiovascular complications [94]. Additional studies have corroborated the finding that PPIs reduce the risk of UGIB, GI ulcers, and erosions in patients on concurrent DAPT [95,96,97].

An additional, very important risk factor for complications in ACS patients that has recently been raised is in-hospital bleeding (IHB). Published in February 2025, the registry study analyzed data from over 23,000 patients collected in the PRAISE registry. A total of 1060 patients experienced IHB during hospitalization, which always resulted in the use of less optimal therapy after discharge. In the group of patients after IHB, a higher overall mortality, a higher rate of major bleeding and recurrent myocardial infarction were observed in the one-year follow-up. For this reason, special attention should be paid to this group of patients during the follow-up after discharge from the hospital [98].

## 6. Conclusions

The safety of using various types of anticoagulant therapy in patients with ACS has been the subject of numerous studies and meta-analyses for many years. However, numerous comparative studies do not clearly indicate which drugs are a safer choice for patients in the context of bleeding complications. Currently, it seems that the proper assessment of the bleeding risk, depending on the patient’s clinical condition, is still more important in the appropriate selection of antithrombotic therapy.

## Figures and Tables

**Table 1:** Antithrombotic drugs used in acute coronary syndromes. LD—loading dose; MD—maintenance dose; CKD—chronic kidney disease; CrCl—creatinine clearance; o.d.—once a day; b.i.d—twice a day; aPTT—activated partial thromboplastin time; ACS—acute coronary syndrome; i.v.—intravenously; s.c.—subcutaneous; PPCI—primary percutaneous coronary intervention; UFH—unfractionated heparin.

Name	Mechanism of Action	Route of Administration	Recommended Dosage
Aspirin	antiplatelet drug COX (TXA_2 inhibitor_)	orally or intravenously	LD 150–300 mg orally or 75–250 mg i.v, followed by an oral MD of 75–100 mg *
Clopidogrel	antiplatelet drug, P2Y12 receptor inhibitor	Orally	LD of 300–600 mg orally, followed by an MD of 75 mg o.d. *
Prasugrel	antiplatelet drug, P2Y12 receptor inhibitor	Orally	LD of 60 mg orally, followed by an MD of 10 mg o.d. *
Ticagrelor	antiplatelet drug, P2Y12 receptor inhibitor	Orally	LD of 180 mg orally, followed by an MD of 90 mg b.i.d. *
Cangrelor	antiplatelet drug, P2Y12 receptor inhibitor	Intravenously	Bolus of 30 mcg/kg i.v. followed by 4 mcg/kg/min infusion for at least 2 h or the duration of the procedure *
Eptifibatide	antiplatelet drug, GP IIb/IIIa receptor inhibitor	Intravenously	Double bolus of 180 mcg/kg i.v. (given at a 10-min interval) followed by an infusion of 2.0 mcg/kg/min for up to 18 h. For CrCl 30–50 mL/min: first LD, 180 mcg/kg i.v. bolus (max 22.6 mg); maintenance infusion, 1 mcg/kg/min (max 7.5 mg/h)
Tirofiban	antiplatelet drug, GP IIb/IIIa receptor inhibitor	intravenously	Bolus of 25 mcg/kg i.v. over 3 min, followed by an infusion of 0.15 mcg/kg/min for up to 18 h. For CrCl ≤60 mL/min: LD, 25 mcg/kg i.v. over 5 min followed by a maintenance infusion of 0.075 mcg/kg/min continued for up to 18 h
UFH	anticoagulant drug	Intravenously	Initial treatment: i.v. bolus 70–100 U/kg followed by i.v. infusion titrated to achieve the aPTT of 60–80 s *
Enoxaparin	anticoagulant drug	Subcutaneously	Initial treatment: for treatment of ACS 1 mg/kg b.i.d. subcutaneously for a minimum of 2 days and continued until clinical stabilization. For CrCl below 30 mL per minute (by Cockcroft–Gault equation), the dosage should be reduced to 1 mg per kg o.d.
Bivalirudin	anticoagulant drug	Intravenously	During PPCI: 0.75 mg/kg i.v. bolus followed by i.v. infusion of 1.75 mg/kg/h for 4 h after the procedure. For CrCl below 30 mL/min (by Cockcroft–Gault equation), maintenance infusion should be reduced to 1 mg/kg/h.
Fondaparinux	Anticoagulant drug	Subcutaneously	Initial treatment: 2.5 mg/d subcutaneously. During PCI: A single bolus of UFH is recommended. Avoid if CrCl < 20 mL/min.

* no specific dose adjustment in CKD patients.

**Table 2:** Summary of studies about intravenous antiplatelet drugs. LMWH—low-molecular-weight heparin.

Study Name	Group Size	Drug	Conclusions	Complications
CHAMPION PLATFORM	5362	Cangrelor	Lower rate of stent thrombosis and all-cause mortality in cangrelor vs. placebo group	No statistical differences in the incidence of bleeding between the cangrelor vs. placebo group
CHAMPION PCI	8877	Cangrelor	No statistical differences in the mortality and myocardial infarction between the cangrelor vs. placebo group	Minor but not major bleeding occurred more often in the cangrelor vs. placebo group
CHAMPION PHOENIX	10,942	Cangrelor	Lower rate of all-cause death, myocardial infarction, ischemia-induced revascularization, or stent thrombosis within 48 h in cangrelor vs. placebo group	Higher risk of major bleeding or transfusion in cangrelor vs. placebo group
EPIC	2099	Abciximab	Reduction of death, nonfatal myocardial infarction, repeat revascularization in abciximab group	Significant increase in both major bleeding and transfusion events in the abciximab group
EPILOG	2792	Abciximab + LMWH	Reduction of death, nonfatal myocardial infarction, repeat revascularization in abciximab + LMWH group	Lower rate of major bleeding in abciximab + low-dose heparin group vs. standard-dose heparin groups
EPISTENT	2399	Abciximab	Reduction of death, nonfatal myocardial infarction, repeat revascularization in abciximab groups	No statistical differences in the incidence of bleeding
CAPTURE	1265	Abciximab	Reduction of death, nonfatal myocardial infarction, repeat revascularization in abciximab group in patients with unstable angina	No statistical differences in the incidence of bleeding
PURSUIT	10,948	Eptifibatide	Decrease of mortality and incidence of myocardial infarction in the group of patients undergoing coronary artery bypass grafting	No statistical differences in the incidence of bleeding
ESPRIT	2064	Eptifibatide	Reduction of death, nonfatal myocardial infarction, repeat revascularization in eptifibatide group	No statistical differences in the incidence of bleeding
IMPACT II	4010	Eptifibatide	Reduction of death, nonfatal myocardial infarction, and repeat revascularization in the eptifibatide group, regardless of the dose	No statistical differences in the incidence of bleeding
PRISM	3232	Tirofiban vs. heparin	Reduction of death, myocardial infarction or recurrent ischemia in the tirofiban group	No statistical differences in the incidence of bleeding
TARGET	5308	Abciximab vs. Tirofiban	Abciximab is superior than tirofiban	No statistical differences in the incidence of bleeding

**Table 3:** Characteristics of fibrinolytic agents.

Name	Types	Plasminogen Activation	Half-Life Time (Min)	References
Streptokinase	serine proteinase (plasminogen activator)	Indirect	15–30	[50]
Urokinase	serine proteinase (plasminogen activator)	Indirect	15	[51]
Staphylokinase	serine proteinase (plasminogen activator)	Indirect	6	[52]
Tissue-type plasminogen activator	serine proteinase (plasminogen activator)	Direct	4–6	[53]
Alteplase	serine proteinase (plasminogen activator)	Direct	16	[54]
Reteplase	serine proteinase (plasminogen activator)	Direct	15–18	[55]
Tenecteplase	serine proteinase (plasminogen activator)	Direct	24	[56]
Duteplase	serine proteinase (plasminogen activator)	Direct	14–16	[57]
Batroxobin	Metalloproteinase (plasmin)	No	360	[58]
Defibrase	Metalloproteinase (plasmin)	No	180–360	[59]
Fibrinogenase for Injection	Metalloproteinase (plasmin)	No	150–250	[60]

**Table 4:** Contraindications to therapy with fibrinolytic drugs.

Absolute
History of intracranial hemorrhage or stroke of unknown cause Ischemic stroke in the last 6 months Injury or neoplasm of the central nervous system Active bleeding Gastrointestinal bleeding in the last 30 days Surgery or injury in the last 14 days
**Relavite**
Organ puncture in the last 24 h Ischemic nerve system/malformation/known atrioventricular malformation Blood pressure above 185/110 mmHg Time since the onset of symptoms of myocardial infarction more than 24 h Time from onset of stroke symptoms over 4.5 h Current use of warfarin with an INR higher than 1.7 Current use of direct oral anticoagulants

**Table 5:** Scores for estimating bleeding risk. BP—blood pressure; CHF—congestive heart failure; NSAIDs—nonsteroidal anti-inflammatory drugs; Hb—hemoglobin; Hct—hematocrit; hs-cTnT—high-sensitivity cardiac troponin T; GDF-15—growth differentiation factor 15; PPI—proton pump inhibitor.

Risk Score	Risk Factors
HAS-BLED	systolic BP > 160 mm Hg; severe renal or hepatic disease; stroke; previous bleeding; labile INR; age > 65; use of antiplatelets or NSAIDs; alcohol excess
CRUSADE	heart rate; systolic BP; Hct; creatinine clearance; sex; signs of CHF at presentation; diabetes mellitus; history of vascular disease
ABC	age; biomarkers (Hb, hs-cTnT, GDF-15 or cystatin C); previous bleeding
ATRIA	anemia; severe renal disease; age ≥ 75; previous bleeding; hypertension
Alfalfa-MB	age > 65; previous bleeding; anemia; vascular disease; no PPI; use of antiplatelets or NSAIDs; use of rivaroxaban
HEMORRHAGES	hepatic/renal disease; ethanol abuse; malignancy; age > 75; low platelets; re-bleeding risk; hypertension; anemia; genetic factors; increased falls risk; stroke
ORBIT	age ≥ 75; reduced Hb/Hct/anemia; previous bleeding; reduced renal function; use of antiplatelets

## Data Availability

No new data were created or analyzed in this study.

## References

[1] Vranckx P., White H.D., Huang Z., Mahaffey K.W., Armstrong P.W., Van de Werf F., Moliterno D.J., Wallentin L., Held C., Aylward P.E. (2016). Validation of BARC bleeding criteria in patients with acute coronary syndromes: The TRACER trial. J. Am. Coll. Cardiol..

[2] Ndrepepa G., Berger P.B., Mehilli J., Seyfarth M., Neumann F.-J., Schömig A., Kastrati A. (2008). Periprocedural bleeding and 1-year outcome after percutaneous coronary interventions: Appropriateness of including bleeding as a component of a quadruple end point. J. Am. Coll. Cardiol..

[3] Pearce S., Maingard J.T., Li K., Kok H.K., Barras C.D., Russell J.H., Hirsch J.A., Chandra R.V., Jhamb A., Thijs V. (2020). Antiplatelet Drugs for Neurointerventions: Part 1 Clinical Pharmacology. Clin. Neuroradiol..

[4] Pelliccia F., Gragnano F., Pasceri V., Cesaro A., Zimarino M., Calabrò P. (2022). Risk Scores of Bleeding Complications in Patients on Dual Antiplatelet Therapy: How to Optimize Identification of Patients at Risk of Bleeding after Percutaneous Coronary Intervention. J. Clin. Med..

[5] Costa F., van Klaveren D., James S., Heg D., Räber L., Feres F., Pilgrim T., Hong M.K., Kim H.S., Colombo A. (2017). Derivation and validation of the predicting bleeding complications in patients undergoing stent implantation and subsequent dual antiplatelet therapy (PRECISE-DAPT) score: A pooled analysis of individual-patient datasets from clinical trials. Lancet.

[6] Natsuaki M., Morimoto T., Yamaji K., Watanabe H., Yoshikawa Y., Shiomi H., Nakagawa Y., Furukawa Y., Kadota K., Ando K. (2018). CREDO-Kyoto PCI/CABG Registry Cohort 2, RESET, and NEXT trial investigators. Prediction of Thrombotic and Bleeding Events After Percutaneous Coronary Intervention: CREDO-Kyoto Thrombotic and Bleeding Risk Scores. J. Am. Heart. Assoc..

[7] D’Ascenzo F., Abu-Assi E., Raposeiras-Roubín S., Henriques J.P., Saucedo J., González-Juanatey J.R., Wilton S.B., Kikkert W.J., Nuñez-Gil I., Ariza-Sole A. (2016). BleeMACS: Rationale and design of the study. J. Cardiovasc. Med..

[8] Zhao X., Li J., Tang X., Xian Y., Jiang L., Chen J., Gao L., Gao Z., Qiao S., Yang Y. (2019). Prognostic Value of the PARIS Thrombotic Risk Score for 2-Year Mortality After Percutaneous Coronary Intervention. Clin. Appl. Thromb. Hemost..

[9] García Rodríguez L.A., Martín-Pérez M., Hennekens C.H., Rothwell P.M., Lanas A. (2016). Bleeding Risk with Long-Term Low-Dose Aspirin: A Systematic Review of Observational Studies. PLoS ONE.

[10] Baigent C., Blackwell L., Collins R., Emberson J., Godwin J., Peto R., Buring J., Hennekens C., Kearney P., Meade T. (2009). Aspirin in the primary and secondary prevention of vascular disease: Collaborative meta-analysis of individual participant data from randomised trials. Lancet.

[11] Gwon H.C., Hahn J.Y., Park K.W., Song Y.B., Chae I.H., Lim D.S., Han K.R., Choi J.H., Choi S.H., Kang H.J. (2012). Six-month versus 12-month dual antiplatelet therapy after implantation of drug-eluting stents: The efficacy of Xience/Promus versus Cypher to reduce late loss after stenting (excellent) randomized, multicenter study. Circulation.

[12] Valgimigli M., Campo G., Monti M., Vranckx P., Percoco G., Tumscitz C., Castriota F., Colombo F., Tebaldi M., Fucà G. (2012). Short- versus long-term duration of dual-antiplatelet therapy after coronary stenting: A randomized multicenter trial. Circulation.

[13] Dewilde W.J., Oirbans T., Verheugt F.W., Kelder J.C., De Smet B.J., Herrman J.P., Adriaenssens T., Vrolix M., Heestermans A.A., Vis M.M. (2013). Use of clopidogrel with or without aspirin in patients taking oral anticoagulant therapy and undergoing percutaneous coronary intervention: An open-label, randomised, controlled trial. Lancet.

[14] Kerneis M., Gibson C.M., Chi G., Mehran R., AlKhalfan F., Talib U., Pahlavani S., Mir M., Bode C., Halperin J.L. (2018). Effect of procedure and coronary lesion characteristics on clinical outcomes among atrial fibrillation patients undergoing percutaneous coronary intervention: Insights from the PIONEER AF-PCI trial. JACC Cardiovasc. Interv..

[15] Cannon C.P., Bhatt D.L., Oldgren J., Lip G.Y.H., Ellis S.G., Kimura T., Maeng M., Merkely B., Zeymer U., Gropper S. (2017). Dual antithrombotic therapy with dabigatran after PCI in atrial fibrillation. N. Eng. J. Med..

[16] Lopes R.D., Heizer G., Aronson R., Vora A.N., Massaro T., Mehran R., Goodman S.G., Windecker S., Darius H., Li J. (2019). Antithrombotic therapy after acute coronary syndrome or PCI in atrial fibrillation. N. Eng. J. Med..

[17] Kubica J., Adamski P., Dobrzycki S., Gajda R., Gąsior M., Gierlotka M., Jaguszewski M., Legutko J., Lesiak M., Navarese E.P. (2024). CangrelorExpanding therapeutic options in patients with acute coronary syndrome. Cardiol. J..

[18] Bhatt D.L., Lincoff A.M., Gibson C.M., Stone G.W., McNulty S., Montalescot G., Kleiman N.S., Goodman S.G., White H.D., Mahaffey K.W. (2009). Intravenous platelet blockade with cangrelor during PCI. N. Eng. J. Med..

[19] Harrington R.A., Stone G.W., McNulty S., White H.D., Lincoff A.M., Gibson C.M., Pollack C.V., Montalescot G., Mahaffey K.W., Kleiman N.S. (2009). Platelet inhibition with cangrelor in patients undergoing PCI. N. Eng. J. Med..

[20] Bhatt D., Stone G., Mahaffey K., Gibson C.M., Steg P.G., Hamm C.W., Price M.J., Leonardi S., Gallup D., Bramucci E. (2013). Effect of platelet inhibition with cangrelor during PCI on ischemic events. N. Eng. J. Med..

[21] Boersma E., Akkerhuis K.M., Théroux P., Califf R.M., Topol E.J., Simoons M.L. (1999). Platelet glycoprotein IIb/IIIa receptor inhibition in non-ST-elevation acute coronary syndromes: Early benefit during medical treatment only, with additional protection during percutaneous coronary intervention. Circulation.

[22] Vergara-Jimenez J., Tricoci P. (2010). Safety and efficacy of abciximab as an adjunct to percutaneous coronary intervention. Vasc. Health Risk Manag..

[23] Califf R.M., Lincoff A.M., Tcheng J.E., Topol E.J. (1995). An overview of the results of the EPIC trial. Eur. Heart J..

[24] EPILOG Investigators (1997). Platelet glycoprotein IIb/IIIa receptor blockade and low-dose heparin during percutaneous coronary revascularization. N. Eng. J. Med..

[25] EPISTENT Investigators (1998). Randomised placebo-controlled and balloon-angioplasty controlled trial to assess safety of coronary stenting with use of platelet glycoprotein-IIb/IIIa blockade. The EPISTENT investigators. Evaluation of platelet IIb/IIIa inhibitor for stenting. Lancet.

[26] Capture Investigators (1997). Randomised placebo-controlled trial of abciximab before and during coronary intervention in refractory unstable angina: The CAPTURE Study. Lancet.

[27] The PURSUIT Investigators (1999). Stroke in patients with acute coronary syndromes. Incidence and outcomes in platelet glycoprotein IIb/IIIa in unstable angina: Receptor suppresion using integrilin therapy (PURSUIT) Trial. Circulation.

[28] (1998). The PRISM Study Investigators: A comparison of aspirin plus tyrofibane with aspirin plus heparin for unstable angina. N. Eng. J. Med..

[29] The ESPRIT Investigators (2000). Novel dosing regimen of eptifibatide in planned coronary stent implantation (ESPRIT): A randomised, placebo-controlled trial. Lancet.

[30] IMPACT-II Investigators (1997). Randomized placebo-controlled trial of effect of eptifibatide on complications of percutaneous coronary intervention: IMPACT-II. Lancet.

[31] Topol E.J., Moliterno D.J., Herrmann H.C., Powers E.R., Grines C.L., Cohen D.J., Cohen E.A., Bertrand M., Neumann F.J., Stone G.W. (2001). Comparison of two platelet glycoprotein IIb/IIIa inhibitors, tirofiban and abciximab, for the prevention of ischemic events with percutaneous coronary revascularization. N. Eng. J. Med..

[32] Kim B.K., Hong M.K., Shin D.H., Nam C.M., Kim J.S., Ko Y.G., Choi D., Kang T.S., Park B.E., Kang W.C. (2012). A new strategy for discontinuation of dual antiplatelet therapy: The RESET trial (REal Safety and Efficacy of 3-month dual antiplatelet Therapy following Endeavor zotarolimus-eluting stent implantation). J. Am. Coll. Cardiol..

[33] Feres F., Costa R.A., Abizaid A., Leon M.B., Marin-Neto J.A., Botelho R.V., King S.B., Negoita M., Liu M., de Paula J.E. (2013). Three vs twelve months of dual antiplatelet therapy after zotarolimus-eluting stents: The OPTIMIZE randomized trial. JAMA.

[34] Hahn J.Y., Song Y.B., Oh J.H., Chun W.J., Park Y.H., Jang W.J., Im E.S., Jeong J.O., Cho B.R., Oh S.K. (2019). Effect of P2Y12 inhibitor monotherapy vs dual antiplatelet therapy on cardiovascular events in patients undergoing percutaneous coronary intervention: The SMART-CHOICE randomized clinical trial. JAMA.

[35] Mehran R., Baber U., Sharma S.K., Cohen D.J., Angiolillo D.J., Briguori C., Cha J.Y., Collier T., Dangas G., Dudek D. (2019). Ticagrelor with or without aspirin in high-risk patients after PCI. N. Eng. J. Med..

[36] Kim B.K., Hong S.J., Cho Y.H., Yun K.H., Kim Y.H., Suh Y., Cho J.Y., Her A.Y., Cho S., Jeon D.W. (2020). Effect of ticagrelor monotherapy vs ticagrelor with aspirin on major bleeding and cardiovascular events in patients with acute coronary syndrome: The TICO randomized clinical trial. JAMA.

[37] Giacoppo D., Matsuda Y., Fovino L.N., D’Amico G., Gargiulo G., Byrne R.A., Capodanno D., Valgimigli M., Mehran R., Tarantini G. (2021). Short dual antiplatelet therapy followed by P2Y12 inhibitor monotherapy vs. prolonged dual antiplatelet therapy after percutaneous coronary intervention with second-generation drug-eluting stents: A systematic review and meta-analysis of randomized clinical trials. Eur. Heart J..

[38] Valgimigli M., Frigoli E., Heg D., Tijssen J., Jüni P., Vranckx P., Ozaki Y., Morice M.C., Chevalier B., Onuma Y. (2021). Dual antiplatelet therapy after PCI in patients at high bleeding risk. N. Eng. J. Med..

[39] Mehran R., Cao D., Angiolillo D.J., Bangalore S., Bhatt D.L., Ge J., Hermiller J., Makkar R.R., Neumann F.J., Saito S. (2021). 3- or 1-month DAPT in patients at high bleeding risk undergoing everolimus-eluting stent implantation. JACC Cardiovasc. Interv..

[40] Hong S.J., Kim J.S., Hong S.J., Lim D.S., Lee S.Y., Yun K.H., Park J.K., Kang W.C., Kim Y.H., Yoon H.J. (2021). 1-month dual-antiplatelet therapy followed by aspirin monotherapy after polymer-free drug-coated stent implantation: One-Month DAPT Trial. JACC Cardiovasc. Interv..

[41] Watanabe H., Domei T., Morimoto T., Natsuaki M., Shiomi H., Toyota T., Ohya M., Suwa S., Takagi K., Nanasato M. (2019). Effect of 1-month dual antiplatelet therapy followed by clopidogrel vs 12-month dual antiplatelet therapy on cardiovascular and bleeding events in patients receiving PCI: The STOPDAPT-2 randomized clinical trial. JAMA.

[42] Costa F., Montalto C., Branca M., Hong S.J., Watanabe H., Franzone A., Vranckx P., Hahn J.Y., Gwon H.C., Feres F. (2023). Dual antiplatelet therapy duration after percutaneous coronary intervention in high bleeding risk: A meta-analysis of randomized trials. Eur. Heart J..

[43] Agbaedeng T.A., Noubiap J.J., Roberts K.A., Chew D.P., Psaltis P.J., Amare A.T. (2024). Sex-Based Outcomes of Dual-Antiplatelet Therapy After Percutaneous Coronary Intervention: A Pairwise and Network Meta-Analysis. Drugs.

[44] Vranckx P., Valgimigli M., Jüni P., Hamm C., Steg P.G., Heg D., van Es G.A., McFadden E.P., Onuma Y., van Meijeren C. (2018). Ticagrelor plus aspirin for 1 month, followed by ticagrelor monotherapy for 23 months vs aspirin plus clopidogrel or ticagrelor for 12 months, followed by aspirin monotherapy for 12 months after implantation of a drug-eluting stent: A multicentre, open-label, randomised superiority trial. Lancet.

[45] Gragnano F., Mehran R., Branca M., Franzone A., Baber U., Jang Y., Kimura T., Hahn J.Y., Zhao Q., Windecker S. (2023). P2Y12 inhibitor monotherapy or dual antiplatelet therapy after complex percutaneous coronary interventions. J. Am. Coll. Cardiol..

[46] Verstraete M., Collen D. (1986). Pharmacology of thrombolytic drugs. J. Am. Coll. Cardiol..

[47] Tang M., Hu C., Lin H., Yan H. (2023). Fibrinolytic drugs induced hemorrhage: Mechanisms and solutions. Blood Coagul. Fibrinol..

[48] Kumar S.S., Sabu A. (2019). Fibrinolytic Enzymes for Thrombolytic Therapy. Adv. Exp. Med. Biol..

[49] Marder V.J., Novokhatny V. (2010). Direct fibrinolytic agents: Biochemical attributes, preclinical foundation and clinical potential. J. Thromb. Haemost..

[50] Baharifar H., Khoobi M., Arbabi Bidgoli S., Amani A. (2020). Preparation of PEG-grafted chitosan/streptokinase nanoparticles to improve biological half-life and reduce immunogenicity of the enzyme. Int. J. Biol. Macromol..

[51] Zhang N., Li C., Zhou D., Ding C., Jin Y., Tian Q., Meng X., Pu K., Zhu Y. (2018). Cyclic RGD functionalized liposomes encapsulating urokinase for thrombolysis. Acta Biomater..

[52] Qi F., Hu C., Yu W., Hu T. (2018). Conjugation with eight-arm PEG markedly improves the in vitro activity and prolongs the blood circulation of staphylokinase. Bioconjug. Chem..

[53] Ichinose A., Takio K., Fujikawa K. (1986). Localization of the binding site of tissue-type plasminogen activator to fibrin. J. Clin. Investig..

[54] LeCouffe N.E., Kappelhof M., Treurniet K.M., Rinkel L.A., Bruggeman A.E., Berkhemer O.A., Wolff L., van Voorst H., Tolhuisen M.L., Dippel D.W.J. (2021). A randomized trial of intravenous alteplase before endovascular treatment for stroke. N. Eng. J. Med..

[55] Chen S., Chen D., Liu Y., Xu Y., Lin H., Cheng Y., Li J., Meng C., Liang M., Yuan C. (2022). Enhanced clot lysis by a single point mutation in a reteplase variant. Br. J. Haematol..

[56] Warach S.J., Dula A.N., Milling T.J. (2020). Tenecteplase thrombolysis for acute ischemic stroke. Stroke.

[57] Mori E., Yoneda Y., Tabuchi M., Yoshida T., Ohkawa S., Ohsumi Y., Kitano K., Tsutsumi A., Yamadori A. (1992). Intravenous recombinant tissue plasminogen activator in acute carotid artery territory stroke. Neurology.

[58] Ding J., Pan L., Hu Y., Rajah G.B., Zhou D., Bai C., Ya J.Y., Wang Z.A., Jin K.X., Guan J.W. (2019). Batroxobin in combination with anticoagulation may promote venous sinus recanalization in cerebral venous thrombosis: A real-world experience. CNS Neurosci. Ther..

[59] Bourgain R.H., Six F. (1975). The effect of defibrase on arterial thrombus formation. Thromb. Res..

[60] Gasmi A., Chabchoub A., Guermazi S., Karoui H., Elayeb M., Dellagi K. (1997). Further characterization and thrombolytic activity in a rat model of a fibrinogenase from vipera lebetina venom. Thromb. Res..

[61] Tillett W.S., Garner R.L. (1933). The Fibrinolytic Activity of Hemolytic Streptococci. J. Exp. Med..

[62] Johnson A.J., Tillett W.S. (1952). The lysis in rabbits of intravascular blood clots by the streptococcal fibrinolytic system (streptokinase). J. Exp. Med..

[63] Sherry S. (1954). The fibrinolytic activity of streptokinase activated human plasmin. J. Clin. Investing.

[64] Fletcher A.P., Alkjaersig N., Smyrniotis F.E., Sherry S. (1958). The treatment of patients suffering from early myocardial infarction with massive and prolonged streptokinase therapy. Trans. Assoc. Am. Physicians.

[65] Ruegsegger P., Nydick I., Hutter R.C., Freiman A.H., Bang N.U., Cliffton E.E., Ladue J.S. (1959). Fibrinolytic (plasmin) therapy of experimental coronary thrombi with alteration of the evolution of myocardial infarction. Circulation.

[66] Gruppo Italiano per lo Studio della Sopravvivenza nell’Infarto Miocardico (1990). GISSI-2: A factorial randomised trial of alteplase versus streptokinase and heparin versus no heparin among 12,490 patients with acute myocardial infarction. Lancet.

[67] Armstrong P.W., Gershlick A.H., Goldstein P., Wilcox R., Danays T., Lambert Y., Sulimov V., Rosell Ortiz F., Ostojic M., Welsh R.C. (2013). Fibrinolysis or primary PCI in ST-segment elevation myocardial infarction. N. Eng. J. Med..

[68] Roule V., Ardouin P., Blanchart K., Lemaitre A., Wain-Hobson J., Legallois D., Alexandre J., Sabatier R., Milliez P., Beygui F. (2016). Prehospital fibrinolysis versus primary percutaneous coronary intervention in ST-elevation myocardial infarction: A systematic review and meta-analysis of randomized controlled trials. Crit. Care.

[69] Ibanez B., James S., Agewall S., Antunes M.J., Bucciarelli-Ducci C., Bueno H., Caforio A.L.P., Crea F., Goudevenos J.A., Halvorsen S. (2018). 2017 ESC Guidelines for the management of acute myocardial infarction in patients presenting with ST-segment elevation: The Task Force for the management of acute myocardial infarction in patients presenting with ST-segment elevation of the European Society of Cardiology (ESC). Eur. Heart J..

[70] Jauch E.C., Saver J.L., Adams H.P., Bruno A., Connors J.J., Demaerschalk B.M., Khatri P., McMullan P.W., Qureshi A.I., Rosenfield K. (2013). Guidelines for the early management of patients with acute ischemic stroke: A guideline for healthcare professionals from the American Heart Association/American Stroke Association. Stroke.

[71] Liu M., Pan Y., Zhou L., Wang Y. (2019). Low-dose rt-PA may not decrease the incidence of symptomatic intracranial haemorrhage in patients with high risk of symptomatic intracranial haemorrhage. Neurol. Res..

[72] Holbrook A., Schulman S., Witt D.M., Vandvik P.O., Fish J., Kovacs M.J., Svensson P.J., Veenstra D.L., Crowther M., Guyatt G.H. (2012). Evidence-based management of anticoagulant therapy: Antithrombotic Therapy and Prevention of Thrombosis, 9th ed: American College of Chest Physicians Evidence-Based Clinical Practice Guidelines. Chest.

[73] Mulloy B., Hogwood J., Gray E., Lever R., Page C.P. (2016). Pharmacology of Heparin and Related Drugs. Pharmacol. Rev..

[74] Warnock L.B., Huang D. (2023). Heparin.

[75] Solari F., Varacallo M. (2025). Low-Molecular-Weight Heparin (LMWH). StatPearls.

[76] Hirsh J., Anand S.S., Halperin J.L., Fuster V. (2001). Mechanism of action and pharmacology of unfractionated heparin. Arterioscler Thromb Vasc Biol..

[77] Walenga J.M., Fareed J., Jeske W.P., Bıck R.L., Samama M.M. (2002). Development of a Synthetic Heparin Pentasaccharide: Fondaparinux. Turk. J. Haematol..

[78] Kumar A., Talwar A., Farley J.F., Muzumdar J., Schommer J.C., Balkrishnan R., Wu W. (2019). Fondaparinux Sodium Compared with Low-Molecular-Weight Heparins for Perioperative Surgical Thromboprophylaxis: A Systematic Review and Meta-analysis. J. Am. Heart. Assoc..

[79] Omerovic E., James S., Råmundal T., Fröbert O., Linder R., Danielewicz M., Hamid M., Pagonis C., Henareh L., Wagner H. (2024). Bivalirudin versus heparin in ST and non-ST-segment elevation myocardial infarction-Outcomes at two years. Cardiovasc. Revasc. Med..

[80] Yan Y., Guo J., Wang X., Wang G., Fan Z., Yin D., Wang Z., Zhang F., Tian C., Gong W. (2024). RIGHT Investigators. Postprocedural Anticoagulation After Primary Percutaneous Coronary Intervention for ST-Segment-Elevation Myocardial Infarction: A Multicenter, Randomized, Double-Blind Trial. Circulation.

[81] Wu Q., Xie T., Chen Y., Zhou Y., Han X. (2024). The Effects of Bivalirudin and Ordinary Heparin on the Incidence of Bleeding Events and the Level of Inflammation after Interventional Therapy for Acute Myocardial Infarction. Altern. Ther. Health Med..

[82] Alturkmani H., Uretsky B., Patel S., Albadaineh M.N., Alqaisi O., Alaiwah M., Cross M., Abbasi D., Rollefson W. (2024). Safety and Efficacy of Enoxaparin During Low-Risk Elective Percutaneous Coronary Intervention. Am. J. Cardiol..

[83] Kumar P., Shaik M., Yuan J. (2017). Choosing between enoxaparin and fondaparinux for the management of patients with acute coronary syndrome: A systematic review and meta-analysis. BMC Cardiovasc. Disord..

[84] Qiao J., Zhang X., Zhang J., Li P., Xu B., Wang S., Jiang H., Shen Y., Wang K. (2016). Comparison between fondaparinux and low-molecular-weight heparin in patients with acute coronary syndrome: A meta-analysis. Cardiology.

[85] Imberti J.F., Mei D.A., Vitolo M., Bonini N., Proietti M., Potpara T., Lip G.Y.H., Boriani G. (2022). Comparing atrial fibrillation guidelines: Focus on stroke prevention, bleeding risk assessment and oral anticoagulant recommendations. Eur. J. Intern. Med..

[86] Bang J., Choi S.Y., Kim M.H., Serebruany V. (2017). CRUSADE Score is Superior to Platelet Function Testing for Prediction of Bleeding in Patients Following Coronary Interventions. eBioMedicine.

[87] Gorog D.A., Gue Y.X., Chao T.F., Fauchier L., Ferreiro J.L., Huber K., Konstantinidis S.V., Lane D.A., Marin F., Oldgren J. (2022). Assessment and mitigation of bleeding risk in atrial fibrillation and venous thromboembolism: A Position Paper from the ESC Working Group on Thrombosis, in collaboration with the European Heart Rhythm Association, the Association for Acute CardioVascular Care and the Asia-Pacific Heart Rhythm Society. Europace.

[88] Lip G.Y., Proietti M., Potpara T., Mansour M., Savelieva I., Tse H.F., Goette A., Camm A.J., Blomstrom-Lundqvist C., Gupta D. (2023). Atrial fibrillation and stroke prevention: 25 years of research at EP Europace journal. Europace.

[89] Shin D.G., Kim S., Kim Y.R. (2022). Bleeding risk in patients with atrial fibrillation treated with combined anti-platelet and non-vitamin K antagonist oral anticoagulant therapy. Rev. Cardiovasc. Med..

[90] Jiménez Díaz V.A., Hovasse T., Íñiguez A., Copt S., Byrne J., Brunel P., Morice M.C., Abizaid A., Tespilli M., Walters D. (2020). Impact of vascular access on outcome after PCI in patients at high bleeding risk: A pre-specified sub-analysis of the LEADERS FREE trial. Rev. Esp. Cardiol..

[91] Borlich M., Zeymer U., Wienbergen H., Hobbach H.P., Cuneo A., Bekeredjian R., Ritter O., Hailer B., Hertting K., Hennersdorf M. (2024). Impact of Access Site on Periprocedural Bleeding and Cerebral and Coronary Events in High-Bleeding-Risk Percutaneous Coronary Intervention: Findings from the RIVA-PCI Trial. Cardiol. Ther..

[92] Piran S., Schulman S. (2019). Treatment of bleeding complications in patients on anticoagulant therapy. Blood.

[93] Bhatt D.L., Cryer B.L., Contant C.F., Cohen M., Lanas A., Schnitzer T.J., Shook T.L., Lapuerta P., Goldsmith M.A., Laine L. (2010). Clopidogrel with or without omeprazole in coronary artery disease. N. Eng. J. Med..

[94] Vaduganathan M., Cannon C.P., Cryer B.L., Liu Y., Hsieh W.H., Doros G., Cohen M., Lanas A., Schnitzer T.J., Shook T.L. (2016). Efficacy and safety of proton-pump inhibitors in high-risk cardiovascular subsets of the COGENT trial. Am. J. Med..

[95] Khan M.Y., Siddiqui W.J., Alvarez C., Aggarwal S., Hasni S.F., Ahmad A., Eisen H. (2018). Reduction in postpercutaneous coronary intervention angina in addition to gastrointestinal events in patients on combined proton pump inhibitors and dual antiplatelet therapy: A systematic review and meta-analysis. Eur. J. Gastroenterol. Hepatol..

[96] Khan S.U., Lone A.N., Asad Z.U., Rahman H., Khan M.S., Saleem M.A., Arshad A., Nawaz N., Sattur S., Kaluski E. (2019). Meta-analysis of efficacy and safety of proton pump inhibitors with dual antiplatelet therapy for coronary artery disease. Cardiovasc. Revasc. Med..

[97] Sehested T.S., Carlson N., Hansen P.W., Gerds T.A., Charlot M.G., Torp-Pedersen C., Køber L., Gislason G.H., Hlatky M.A., Fosbøl E.L. (2019). Reduced risk of gastrointestinal bleeding associated with proton pump inhibitor therapy in patients treated with dual antiplatelet therapy after myocardial infarction. Eur. Heart J..

[98] Spadafora L., Betti M., D’Ascenzo F., De Ferrari G., De Filippo O., Gaudio C., Collet C., Sabouret P., Agostoni P., Zivelonghi C. (2022). Impact of In-Hospital Bleeding on Post-Discharge Therapies and Prognosis in Acute Coronary Syndromes. J. Cardiovasc. Pharmacol..

